# Chiari malformation type 1 presenting as unilateral progressive foot drop: a case report and review of literature

**DOI:** 10.1186/s12887-018-1028-8

**Published:** 2018-02-07

**Authors:** Chamara Jayamanne, Lakkumar Fernando, Sachith Mettananda

**Affiliations:** 1 North Colombo Teaching Hospital, Ragama, Sri Lanka; 2 Colombo, Sri Lanka; 3 District General Hospital, Negombo, Sri Lanka; 4 University of Kelaniya, Kelaniya, Sri Lanka

**Keywords:** Unilateral foot drop, Syringomyelia, Chiari malformation

## Abstract

**Background:**

Foot drop is a disabling clinical condition with multiplicity of causes, which requires detailed evaluation to identify the exact aetiology. Here, we report an extremely rare cause of foot drop in a child, which if not recognized early, could lead to multiple complications.

**Case presentation:**

A 6-year-old girl presented with difficulty in walking and left sided foot droop for1-month duration. On examination she had reduced muscle power in dorsiflexors and plantar flexors and diminished knee and absent ankle jerk in the left side. Sensory loss was noted in L4 and L5 dermatomes on the left side. Superficial abdominal reflex was absent on the left side while preserved in the right. Nerve conduction and electromyography revealed nerve root or spinal cord cause for the foot drop. These results prompted ordering MRI spine and brain which revealed Chiari malformation type-1 with holocord syrinx extending from the cervicomedullary junction to conus medullaris.

**Conclusions:**

This case highlights the importance of considering broad differential diagnosis for foot drop and value of the complete neurological examination including superficial reflexes in arriving at a diagnosis. Prompt diagnosis helped to early neurosurgical referral and intervention which is an important prognostic factor.

## Background

Foot drop is a disabling neurological condition which requires careful evaluation to identify the cause. Differential diagnosis for foot drop is broad however, mostly involve disorders of the peripheral nervous system. Here, we present an extremely rare central cause of foot drop in a child, which if not recognized early, could lead to multiple complications.

## Case presentation

A 6-year-old girl presented with progressively worsening difficulty in walking and left sided foot droop for1-monthduration. There was no pain, paresthesia or sensory symptoms in limbs and she did not complain of headache or backache. There was no history of trauma. She is the second child of non-consanguineous parents and had an uncomplicated birth and perinatal period. She was apparently well except for frequently relapsing nephrotic syndrome for which she was on 5 mg of prednisolone and 60 mg of levimasole on alternative days. Her last (fourth) relapse was six months ago.

On examination she was averagely built for her age. Nervous system examination revealed wasting of anterior compartment of the left leg and reduced muscle power in distal muscle groups on the left side - 0/5 in dorsiflexors and 3/5 in plantar flexors. Muscle power in proximal muscles of the left lower limb, all muscle groups in the right lower limb and both upper limbs were normal (5/5). Left knee jerk was diminished and left ankle jerk and left plantar response were absent. Sensory loss was noted in L4 and L5 dermatomes on the left side. Superficial abdominal reflex was present on the right side however was absent on the left side which pointed towards a central cause for foot drop. All other systems including blood pressure were clinically normal.

**Fig. 1:**
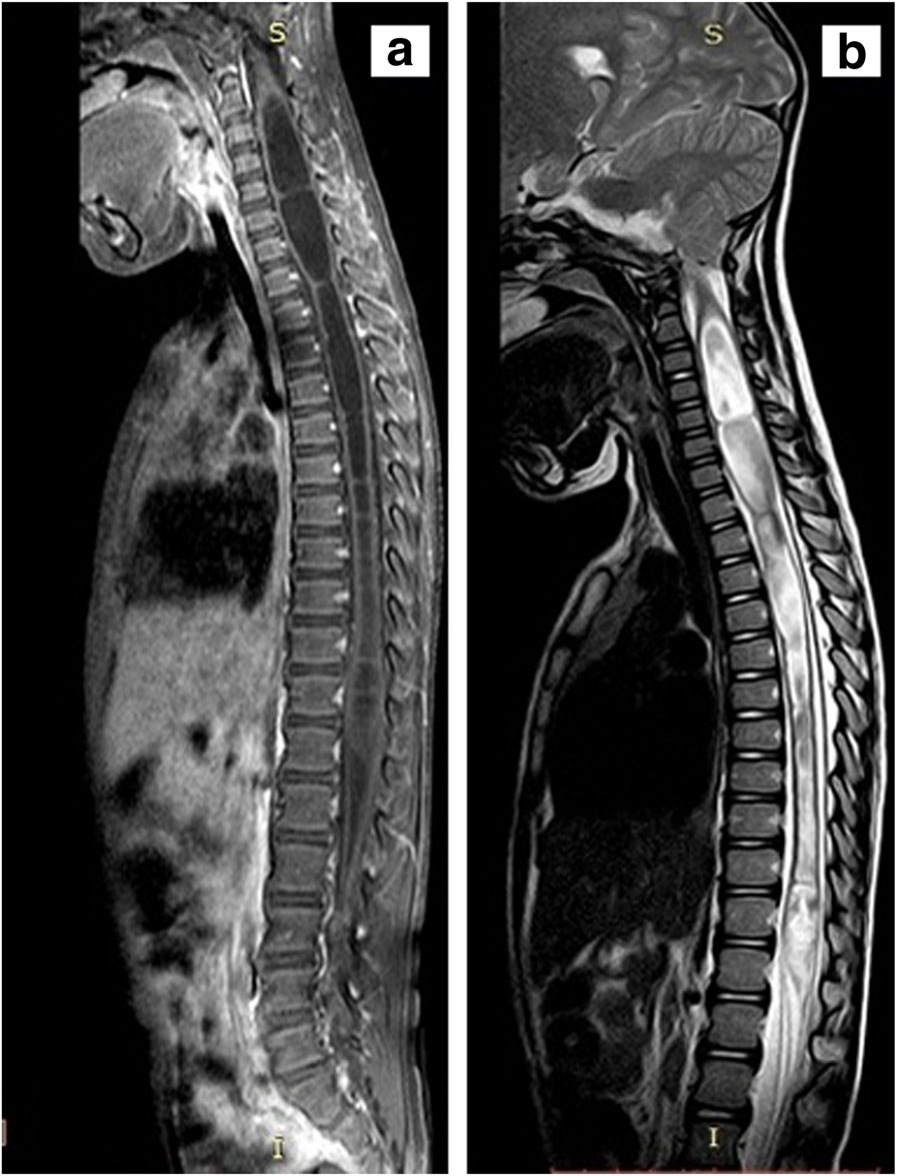
**a** Sagittal T1-weighted sequence of MRI of thoracolumbar spine showing hypointense central cavitary lesion involving whole cord up to conus medullaris. **b** Sagittal T2-weighted sequence of MRI of thoracolumbar spine showed central cavitary lesion extending down to the conus medullaris.

**Fig. 2:**
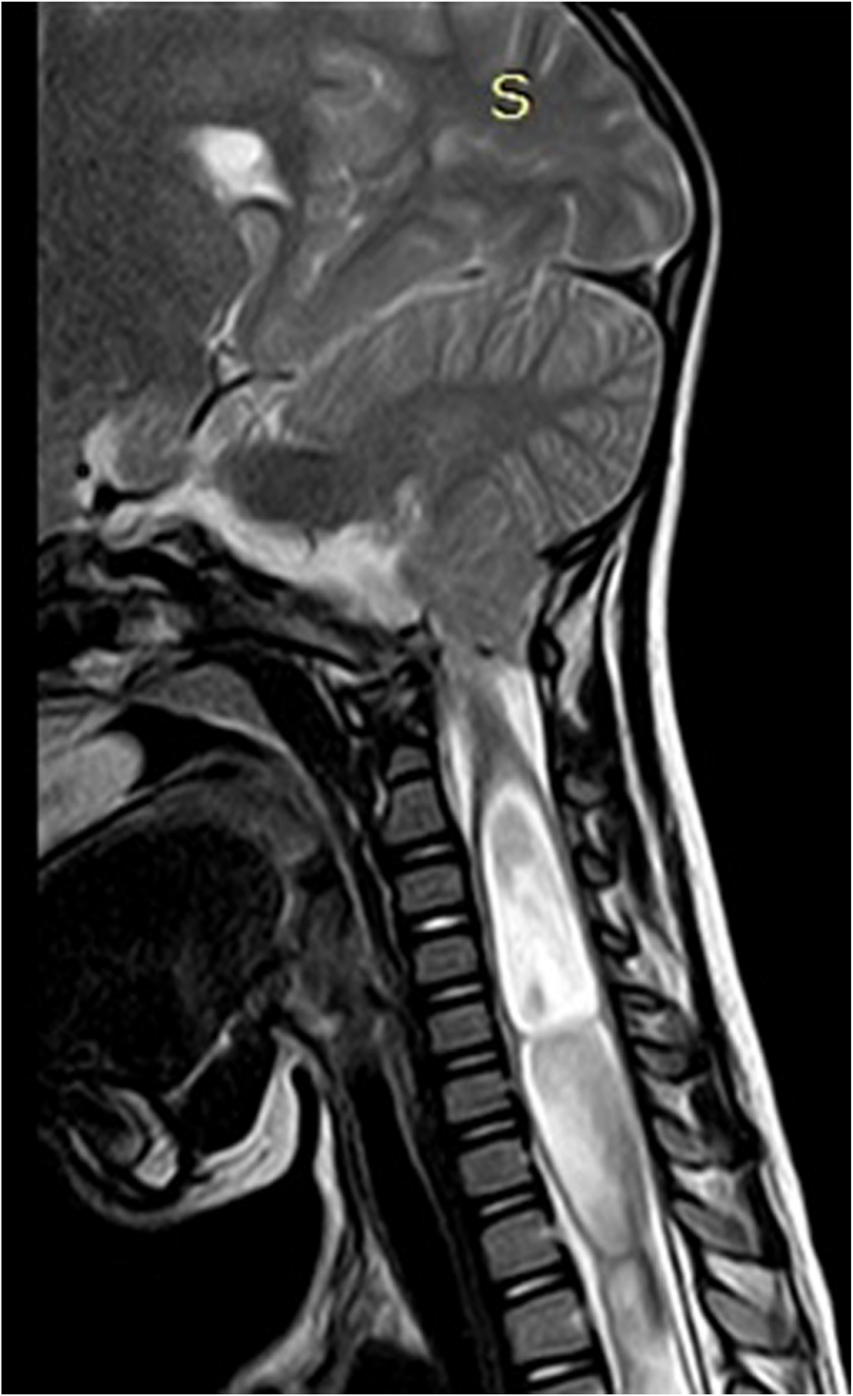
Sagittal T2-weighted MRI of cranio-vertebral junction and cervical spine showing tonsillar herniation and central cavitary lesion suggestive of Chiari malformation type 1 and syringomyelia

Basic investigation including full blood count, erythrocyte sedimentation rate and C-reactive protein were unremarkable and rheumatoid factor and antinuclear antibodies were negative. Nerve conduction studies revealed normal distal amplitude, latency and velocities in common fibular nerve (tested at extensor digitorum brevis) in the left (amplitude 3.3 mV; latency 2.8 ms; velocity 59 m/s) and right (amplitude 2.9 mV; latency 3.1 ms) sides and tibial nerve (tested at abductor hallucis)in the left side(amplitude 20.8 mV; latency 4.2 ms). Sural nerve sensory response was normal. Electromyography (EMG) at left tibialis anterior revealed fibrillations and scanty motor unit potentials (MUP). EMG of left medial gastrocnemius and right tibialis anterior were normal. These results suggested prompt imaging of the spine. MRI of the lumbosacral spine was arranged and was later expanded to whole spine and brain. This revealed a holocord syrinx extending from the cervicomedullary junction to conus medullaris (Fig. 1A and B) and Chiari malformation type 1 without bony deformities (Fig. 2).

The patient was referred to the neuro-surgical team and is awaiting surgery which will include suboccipital craniectomy, C1 laminectomy and duroplasty decompressing foramen magnum. Neuro-rehabilitation was commenced and an ankle foot orthosis was offered for the foot drop.

## Discussion and conclusions

Identification of the exact cause is the most important component of the management of foot drop. Causes are diverse, consists of a broad range of pathologies however, lesions in the peripheral nervous system which include, common peroneal neuropathy, L5 radiculopathy, lumbosacral plexopathy, hereditary neuropathies, mononeuritis multiplex and anterior horn cell diseases predominate. Rarely, central lesions including brain tumours and tuberculomas can cause foot drop. In this report we have described an extremely rare presentation of a central lesion-Chiari malformation type 1- presenting as unilateral foot drop.

Chiari malformations refer to a spectrum of congenital hind brain abnormalities affecting structural relationships between the cerebellum, brain stem, the upper cervical cord and the bony cranial base [1]. Chiari malformation type 1 is the most common form which usually presents with recurrent headache, neck pain, urinary frequency, and progressive lower-extremity spasticity during adolescence or adulthood. It is characterized by displacement of cerebellar tonsils below the level of the foramen magnum which results in impaction of the foramen magnum, compression of the cervicomedullary junction by the herniating tonsils and interruption of the cerebrospinal fluid (CSF) through the region [2, 3]. Disordered CSF flow may result in syringomyelia which usually presents with ‘central cord symptoms’ - numbness followed by the development of atrophy and weakness in the upper extremities. It can have a combination of upper motor and lower motor neuron lesion feature [4, 5].

Our patient did not demonstrate any of the usual presenting symptoms or signs of Chiari malformation type 1 or syringomyelia. Instead, the only presenting clinical features were abnormality in gait and progressive unilateral foot drop. The initial clinical examination and nerve conduction test suggested a lumbar spine pathology however ipsilateral absence of superficial abdominal reflexes suggested a lesion at the thoracic level or above. MRI scan revealed the diagnosis of holocord syrinx with underlying Chiari malformation type 1.

Chiari malformation type 1 and associated holocord syrinx presenting as foot drop is extremely rare and only few case reports are found in the literature (Table 1). The limited number of patients who had presented with foot drop demonstrates variable physical signs and has marked diversity in neurophysiological abnormalities suggesting variable sites of involvement.

**Table 1:** Clinical and neurophysiological findings of previous case reports which describe syringomyelia presenting as foot drop

			Panda AK et al. [8]	Saifudheen K et al. [9]	McMillan HJ et al. [10]		Narry Muhn et al. [11]	Ilya Laufer et al. (Case 2) [12]	Patient described in this case report
					Case 1	Case 2			
Age at presentation (years)			16	14	5	4.5	5.5	9	6
Main complaint			Rapidly progressive right sided foot drop for 2 months	Rapidly progressive bilateral foot drop for 1 week	Left foot drop for 2 months	Abrupt onset right foot drop	Rapidly progressive left foot drop	Right foot weakness for 1 month	Rapidly progressive left foot drop for 1 month
Muscle power (Affected limb) Out of 5	Hip	Flexors	5	N/C	5	N/C	Normal	5	5
		Extensors	5	N/C	5	N/C	Normal	5	5
	Ankle	Dorsi-flexion	0	N/C	0	1	Weak	5	0
		Plantar flexion	3	N/C	4+	N/C	Normal	2	3
Reflex	Knee		Diminished	Diminished	Normal	Absent	Absent	Absent	Diminished
	Ankle		Absent	Diminished	Absent	Absent	Diminished	Diminished	Absent
Nerve conduction	Common fibular nerve	Latency	3 mV	Normal	4.2 mV	5.4 mV	Normal	N/C	2.8 mV
		Amplitude	2.6 ms	Low	1.5 ms	2.6 ms	low	N/C	3.3 ms
		Velocity	43.1 m/s	Normal	44 m/s	45 m/s	Normal	N/C	59 m/s
	Tibial nerve		Normal	Normal	Normal	Normal	Normal	N/C	Normal
Electromyography			Fibrillation waves in right TA, PL, MG, GM	Fibrillation waves in TA, MG	Fibrillation waves in right TA, PL, MG, GM	Active denervation of TA	Fibrillation waves in TA, TP, MG	N/C	Fibrillation waves in left TA. Left MG was Normal

Note: *TA* Tibialis Anterior, *PL* Peronius Longus, *MG* Medial Gastrocnemius, *GM* Gluteus Medius, *TP* Tibialis Posterior, *N/C* Not Commented

Early diagnosis of Chiari malformation type-1 is pivotal as prognosis after the surgery depends on the extent of the neurological deficit prior to the surgery. Our patient is awaiting surgery and has an excellent prognosis [6, 7]. In conclusion, this case highlights the importance of considering broad differential diagnosis for foot drop and value of the complete neurological examination including superficial reflexes in arriving at a diagnosis.

## Abbreviations

MUP: Motor unit potentials
EMG: Electromyography
CSF: Cerebrospinal fluid
TA: Tibialis Anterior
PL: Peronius Longus
MG: Medial Gastrocnemius
GM: Gluteus Medius
TP: Tibialis Posterior
N/C: Not Commented
